# p53 codon 72 polymorphism and breast cancer risk: A meta-analysis

**DOI:** 10.3892/etm.2013.1019

**Published:** 2013-03-20

**Authors:** JING HOU, YUAN JIANG, WENRU TANG, SHUTING JIA

**Affiliations:** 1 Laboratory of Molecular Genetics of Aging and Tumor, Medical Faculty;; 2 Laboratory of Molecular Genetics of Aging and Tumor, Faculty of Life Science and Technology, Kunming University of Science and Technology, Kunming, Yunnan 650500, P.R. China

**Keywords:** p53, meta-analysis, breast cancer

## Abstract

p53 is a tumor suppressor gene and plays important roles in the etiology of breast cancer. Studies have produced conflicting results concerning the role of p53 codon 72 polymorphism (G>C) on the risk of breast cancer; therefore, a meta-analysis was performed to estimate the association between the p53 codon 72 polymorphism and breast cancer. Screening of the PubMed database was conducted to identify relevant studies. Studies containing available genotype frequencies of the p53 codon 72 polymorphism were selected and a pooled odds ratio (OR) with 95% confidence interval (CI) was used to assess the association. Sixty-one published studies, including 28,539 breast cancer patients and 32,788 controls were identified. The results suggest that variant genotypes are not associated with breast cancer risk (Pro/Pro + Arg/Pro vs. Arg/Arg: OR=1.016, 95% CI=0.931–1.11, P=0.722). The symmetric funnel plot, Egger’s test (P=0.506) and Begg’s test (P=0.921) were all suggestive of the lack of publication bias. This meta-analysis suggests that the p53 codon 72 Pro/Pro + Arg/Pro genotypes are not associated with an increased risk of breast cancer. To validate the association between the p53 codon 72 polymorphism and breast cancer, further studies with larger numbers of participants worldwide are required.

## Introduction

Breast cancer is one of the most common cancers affecting the morbidity and mortality of females worldwide (1). While numerous risk factors for breast cancer have been identified, including genetic predisposition and estrogen level, the molecular mechanisms related to breast carcinogenesis remain under analysis (2,3). Previous studies have shown alterations in cell cycle regulatory proteins in breast carcinoma, including the overexpression and increase of the cyclin genes, inactivation and deletions of the Rb gene and alterations of the p53 gene (4–6). Therefore, this disease is a result of collective alterations of oncogenes and tumor suppressor genes. It is well-known that p53, the guardian of the genome, is a stress response protein. p53 functions mainly as a tetramer transcription factor that regulates a large number of genes in response to various stresses, including ontogeny activation and DNA damage (7). p53 is involved in the pro-survival response of cell cycle arrest and DNA damage repair, as well as the pro-death response of apoptosis (8). In the case of a mutation occurring in the p53 gene, p53 may not only lose its normal functions, but also gain new abilities that promote tumorigenesis (9). p53 is the most frequently mutated gene in human tumors; >50% of tumors harbor mutations in the p53 gene (10). Besides its role as a tumor suppressor gene, aberrant p53 expression may play a significant role in regulating angiogenesis (11,12). Chromosomal aberrations and p53 protein abnormalities may be involved in malignant transformation of endometriosis in the ovary (13).

The p53 tumor suppressor gene contains 11 exons, located on chromosome 17p13. The codon 72 polymorphism (rs1042522) is located in exon 4 with a CGC to CCC transition, leading to an arginine to proline amino acid substitution in amino acid position 72 (Arg72Pro). Studies have reported that the codon 72 polymorphism is associated with a risk for the development of cancer (14). The two polymorphic variants have been shown to have not only structural differences, as reflected by distinct electrophoresis patterns of migration, but also different biological properties (15,16). A number of case-control studies have been conducted to explore the correlation between the p53 codon 72 polymorphism and breast cancer risk in humans. However, the results are inconsistent. Another problem is that these published studies have only modest sample sizes, which limits their significance. By performing a meta-analysis, a prevailing method for the quantitative summary of different results, the data may be assessed and the sample size increased to a reasonable level. In the present study, a meta-analysis was conducted to quantitatively assess the effect of the p53 codon 72 polymorphism on the risk of breast cancer.

## Materials and methods

### Publication search

PubMed was searched using the terms ‘p53’, ‘polymorphism’ and ‘breast cancer’ (the last search update was on May 1, 2012). The search was limited to English-language papers. Additional studies were identified by a manual search of the references of original studies. Of the studies with the same or overlapping data published by the same investigators, the most recent ones with the largest number of subjects were selected. Case-control studies containing available genotype frequencies of Arg72Pro were selected.

### Statistical analysis

For the control group of each study, the allelic occurrence was considered and the observed genotype frequencies of the p53 codon 72 polymorphism were assessed for Hardy-Weinberg equilibrium using the *χ*^2^ test. The power of the correlation between the p53 codon 72 polymorphism and breast cancer risk was assessed by odds ratios (ORs) with 95% confidence intervals (CIs). The risks of breast cancer for the GC and CC genotypes, relative to the wild-type GG homozygote were assessed; then, the risks of breast cancer for GC/CC vs. GG and CC vs. GC/GG, and finally the supercilious dominant and recessive effects of the variant C allele were determined. Stratified analyses according to background, the source of controls and clinicopathological individuality were also performed. In considering the possibility of heterogeneity across the studies, an arithmetical test for heterogeneity was performed based on the Q-test. P<0.05 for the Q-test was considered to indicate a lack of heterogeneity among the studies. The summary OR estimate of each study was calculated by the random effects model (17,18). The potential for publication bias was examined by Begg’s test and Egger’s linear regression test. P<0.05 was considered to indicate a statistically significant difference (19). All statistical analyses were performed with Stata software (version 9.0; Stata Corporation, College Station, TX, USA).

## Results

Sixty-one case-control studies concerning the association between p53 codon 72 polymorphism and breast cancer were identified, which included 28,539 breast cancer cases and 32,788 controls. These data were used in a meta-analysis (Table I). The sharing of genotypes in the controls of all the studies was in agreement with Hardy-Weinberg equilibrium.

The results of the association between the p53 codon 72 polymorphism and breast cancer and the heterogeneity test are shown in Table II. The dominant model (Pro/Pro + Pro/Arg vs.Arg/Arg) demonstrated no significant association in Asian (OR=1.028, 95% CI=0.879–1.201, P=0.732), Caucasian (OR=1.036, 95% CI=0.927–1.159, P=0.531) or other subjects (OR=1.016, 95% CI=0.931–1.11, P=0.722).

## Discussion

Given the important roles of p53 in multiple cellular functions, including gene transcription, DNA repair and apoptosis, it is biologically plausible that p53 polymorphisms may be associated with a risk of breast cancer (20). Human breast cancer is a disease with significant clinical consequences. The mechanism of breast cancer remains relatively unknown. Single nucleotide polymorphisms (SNPs) are used as a tool to investigate genetic variations and disease susceptibility.

Although a number of previous studies have reported a significant association between the p53 codon 72 polymorphism and breast cancer risk (21–34), others have identified no such association (35–39). In order to resolve this conflict, in the current study, a meta-analysis was conducted to examine the association between a commonly studied p53 polymorphism (codon 72 G<C, Arg72Pro) and breast cancer risk. A total of 28,539 breast cancer cases and 32,788 controls from 61 studies were included in the final analysis, to derive a more precise estimation of the presence or absence of this association. The polymorphism in codon 72 of the p53 gene was identified to have no association with breast cancer risk, either when the incorporated study populations were pooled or when they were subjected to a stratified analysis consistent with background or the source of controls. The latter result suggests that differences in genetic education, living environment and sources of controls do not impact any potential association between the p53 codon 72 polymorphism and breast cancer risk. Two assets of the current study were the large number of samples included and its failure to identify a significant association in any of the genetic models tested. Nevertheless, several limitations must be acknowledged. The controls in the studies were not homogenously defined, such that the control subjects in the different studies have varying risks of evolving breast cancer. Additionally, the results obtained in the present study are based on unadjusted estimations. A more accurate analysis could be conducted if more detailed individual data were available to allow it to be adjusted according to other covariates, including premenopause, postmenopause, smoking and drinking status, basal metabolic index, family history and environmental factors.

In conclusion, this meta-analysis, with a large model size, provides a strong indication that the p53 codon 72 polymorphism is not associated with breast cancer risk. Future studies should extend this investigation by incorporating other potential risk factors for breast cancer.

## Figures and Tables

**Table I:** Distribution of the p53 codon 72 polymorphism for cases and controls.

			Breast cancer			Control			
Population	First author (ref)	Year	Arg/Arg	Arg/Pro	Pro/Pro	Arg/Arg	Arg/Pro	Pro/Pro	P-value^a^
Asian	Kawajiri (40)	1993	5	51	37	38	165	144	0.36
	Khaliq (41)	2000	13	18	10	177	321	191	0.08
	Li (30)	2002	6	11	11	14	26	10	0.74
	Huang (28)	2003	36	100	64	30	138	114	0.21
	Katiyar (29)	2003	6	51	20	8	24	9	0.27
	Mahasneh (42)	2004	8	19	16	29	51	56	0.01
	Noma (43)	2004	29	69	93	31	76	111	0.00
	Siddique (44)	2005	20	38	36	38	120	107	0.64
	Ma (45)	2006	77	178	149	100	222	150	0.29
	Gochhait (26)	2007	48	109	86	97	160	76	0.52
	Khadang (37)	2007	29	109	83	40	90	75	0.17
	Rajkumar (46)	2008	59	125	66	141	224	135	0.02
	Zhang (34)	2007	17	45	21	33	87	47	0.52
	Lum (47)	2008	88	200	105	13	38	29	0.93
	Singh (48)	2008	13	45	46	12	64	29	0.01
	Kazemi (49)	2009	6	30	6	0	45	12	0.00
	Song (50)	2009	221	544	339	220	508	349	0.16
	Koh (51)	2011	102	197	73	179	319	145	0.90
	Kara (52)	2010	105	84	14	72	80	17	0.44
	Leu (53)	2011	71	90	78	104	129	88	0.00
Caucasian	Själander (22)	1996	24	93	95	61	253	375	0.06
	Weston (33)	1997	6	27	32	3	42	72	0.28
	Wang-Gohrke (54)	1998	5	46	56	21	117	167	0.93
	Papadakis (55)	2000	12	10	34	6	41	12	0.00
	Wang-Gohrke (32)	2002	49	221	282	40	203	300	0.49
	Buyru (25)	2003	12	39	64	12	43	21	0.20
	Suspitsin (56)	2003	42	203	284	27	159	207	0.63
	Menzel (57)	2004	30	170	275	30	114	158	0.17
	Kalemi (23)	2005	3	13	26	9	32	10	0.07
	Ohayon (58)	2005	3	40	89	19	94	54	0.02
	Tommiska (39)	2005	109	617	825	52	278	403	0.67
	Baynes (35)	2007	148	768	1107	166	854	1177	0.52
	Garcia-Closas (59)	2007	196	1021	1368	228	1249	1774	0.69
	Franeková (60)	2007	8	34	49	9	55	92	0.84
	Johnson (61)	2007	30	185	257	183	925	1354	0.15
	Schmidt (38)	2007	618	3228	4499	511	2677	3661	0.48
	Sprague (31)	2007	100	644	909	129	704	1021	0.61
	Akkiprik (24)	2009	20	50	25	12	49	46	0.85
	Cavallone (62)	2008	10	67	80	9	46	57	0.95
	Costa (63)	2008	25	86	137	54	212	380	0.00
	De Vecchi (64)	2008	15	150	185	14	131	207	0.23
	Nordgard (65)	2008	5	58	46	14	34	73	0.00
	Lång (66)	2009	6	45	65	5	58	79	0.15
	Denisov (77)	2009	25	124	148	29	99	147	0.05
	Henríquez (27)	2009	8	54	73	28	100	167	0.03
	Hrstka (68)	2009	40	15	62	45	8	55	0.00
	Bisof (69)	2010	11	23	61	5	42	61	0.51
	Ebner (70)	2010	17	108	138	14	103	137	0.34
	Kara (52)	2010	14	84	105	17	80	72	0.44
	Alshatwi (71)	2012	22	52	26	32	51	17	0.66
	Alawadi (72)	2011	81	200	7	50	112	26	0.00
Others	Weston (33)	1997	1	9	6	4	14	12	0.98
	Weston (73)	1994	7	8	3	12	16	10	0.34
	Helland (74)	1998	6	40	63	13	90	122	0.50
	Mabrouk (36)	2003	3	9	18	4	26	19	0.23
	Damin (21)	2006	64	48	6	70	111	21	0.02
	Cox (75)	2007	104	569	804	131	838	1255	0.57
	Gaudet (76)	2008	46	244	288	34	138	218	0.08
	Aoki (67)	2009	3	29	40	7	53	30	0.01

a P-value for Hardy-Weinberg equilibrium in the control group.

**Table II:** ORs and 95% CI for breast cancer and the p53 codon 72 polymorphism under different genetic models.

Genetic model	Population	Pooled OR (95% CI)	P-value	P-value		
				Heterogeneity	Begg’s test	Egger’s test
Additive (Pro vs. Arg)	Asian	1.016 (0.958–1.077)	0.539	<0.001	0.948	0.889
	Caucasian	1.002 (0.972–1.033)	0.903	<0.001	0.368	0.417
	Others	0.956 (0.88–1.039)	0.288	<0.001	0.463	0.388
	Overall	1 (0.975–1.026)	0.993	<0.001	0.356	0.357
Recessive (Pro/Pro vs. Arg-carriers)	Asian	1.012 (0.882–1.162)	0.861	0.01	0.846	0.862
	Caucasian	1.019 (0.916–1.134)	0.726	<0.001	0.486	0.602
	Others	1.168 (0.852–1.602)	0.335	<0.001	1	0.356
	Overall	1.029 (0.95–1.115)	0.479	<0.001	0.602	0.37
Dominant (Pro-carriers vs. Arg/Arg)	Asian	1.028 (0.879–1.201)	0.732	0.012	0.506	0.921
	Caucasian	1.036 (0.927–1.159)	0.531	0.035	0.773	0.599
	Others	0.912 (0.651–1.277)	0.591	0.064	0.835	0.299
	Overall	1.016 (0.931–1.11)	0.722	0.001	0.565	0.36
Pro/Arg vs. Arg/Arg	Asian	1.027 (0.887–1.188)	0.725	0.082	0.916	0.931
	Caucasian	1.045 (0.926–1.179)	0.473	0.028	0.959	0.868
	Others	0.884 (0.652–1.199)	0.428	0.16	0.835	0.567
	Overall	1.018 (0.933–1.111)	0.689	0.007	0.904	0.739
Pro/Pro vs. Arg/Arg	Asian	1.035 (0.843–1.272)	0.74	0.001	0.248	0.829
	Caucasian	1.029 (0.881–1.203)	0.717	<0.001	0.444	0.667
	Others	1.021 (0.673–1.55)	0.922	0.042	0.345	0.377
	Overall	1.028 (0.916–1.153)	0.639	<0.001	0.188	0.385

OR, odds ratio; CI, confidence interval.
